# Survival of Critically Ill COVID-19 Patients in Sweden During the First Two and a Half Years of the Pandemic*

**DOI:** 10.1097/CCM.0000000000006271

**Published:** 2024-03-28

**Authors:** Ailiana Santosa, Jonatan Oras, Huiqi Li, Chioma Nwaru, Brian Kirui, Fredrik Nyberg

**Affiliations:** 1 School of Public Health and Community Medicine, Institute of Medicine, Sahlgrenska Academy, University of Gothenburg, Gothenburg, Västra Götaland, Sweden.; 2 Department of Anaesthesiology and Intensive Care, Institute of Clinical Sciences, Sahlgrenska Academy, University of Gothenburg, Gothenburg, Västra Götaland, Sweden.; 3 Department of Anaesthesiology and Intensive Care, Region Västra Götaland, Sahlgrenska University Hospital, Gothenburg, Västra Götaland, Sweden.; 4 Population Health Sciences Institute, Faculty of Medical Sciences, Newcastle University, Newcastle upon Tyne, United Kingdom.

**Keywords:** COVID-19, intensive care unit survival, intervention and procedures, medications, variant of concern

## Abstract

**OBJECTIVES:**

Some studies have examined survival trends among critically ill COVID-19 patients, but most were case reports, small cohorts, and had relatively short follow-up periods. We aimed to examine the survival trend among critically ill COVID-19 patients during the first two and a half years of the pandemic and investigate potential predictors across different variants of concern periods.

**DESIGN:**

Prospective cohort study.

**SETTING:**

Swedish ICUs, between March 6, 2020, and December 31, 2022.

**PATIENTS:**

Adult COVID-19 ICU patients of 18 years old or older from the Swedish Intensive Care Register (SIR) that were linked to multiple other national registers.

**MEASUREMENT AND MAIN RESULTS:**

Survival probability and predictors of COVID-19 death were estimated using Kaplan-Meier and Cox regression analysis. Of 8975 patients, 2927 (32.6%) died. The survival rate among COVID-19 critically ill patients appears to have changed over time, with a worse survival in the Omicron period overall. The adjusted hazard ratios (aHRs) comparing older and younger ages were consistently strong but slightly attenuated in the Omicron period. After adjustment, the aHR of death was significantly higher for men, older age (40+ yr), low income, and with comorbid chronic heart disease, chronic lung disease, impaired immune disease, chronic renal disease, stroke, and cancer, and for those requiring invasive or noninvasive respiratory supports, who developed septic shock or had organ failures (*p* < 0.05). In contrast, foreign-born patients, those with booster vaccine, and those who had taken steroids had better survival (aHR = 0.87; 95% CI, 0.80–0.95; 0.74, 0.65–0.84, and 0.91, 0.84–0.98, respectively). Observed associations were similar across different variant periods.

**CONCLUSIONS:**

In this nationwide Swedish cohort covering over two and a half years of the pandemic, ICU survival rates changed over time. Older age was a strong predictor across all periods. Furthermore, most other mortality predictors remained consistent across different variant periods.

**KEY POINTS**

**Question**: We sought to assess temporal trends in survival rate and predictors associated with mortality among COVID-19 ICU patients across different variant of concern (VOC) periods over the first two and a half years.

**Findings**: In this Swedish prospective cohort study, the ICU survival rate has changed over time. Older age was a strong predictor across all periods. After adjusting for baseline characteristics, most mortality predictors remained consistent and significant across VOC periods.

**Meaning**: Lower survival rate was observed among COVID-19 ICU patients in the Omicron period, with age, comorbidities, restricted treatment, and required invasive mechanical ventilation during ICU stay being strong predictors.

During the pandemic, various variants of SARS-CoV-2 have been identified, including Alpha, Beta, Gamma, Delta, and Omicron (1, 2). These variants may increase the virus’ transmissibility, severity, and ability to evade vaccination antibodies (3). Delta variants were more transmissible and associated with higher hospitalization rates, ICU stays, and risk of death than other variants (4–7). The Omicron variant was extremely transmissible (2) but associated with a lower risk of adverse outcomes in hospitalized patients than the Delta variant (8). Several factors have been identified as predictors of COVID-19-related ICU admission and death, including older age, male gender (9, 10), hypertension, diabetes, cardiac disease, chronic kidney disease, liver disease (11–13), and medications (14).

Studies on COVID-19 ICU patients have mainly been conducted in selected healthcare facilities as case reports or small cohorts with relatively short follow-up periods (12, 15–17). Prior Swedish studies have investigated the risk of death related to COVID-19 after ICU care only in the early pandemic (10, 14, 18, 19), effects on ICU resources (20), and comorbidities associated with ICU admission (21, 22). There is limited evidence regarding the mortality trend and its predictors among COVID-19 ICU patients across various variants. This study examines survival trends among critically ill COVID-19 patients during the first two and a half years of the pandemic and investigates potential predictors across different variant of concern (VOC) periods. We hypothesized an improving survival trend, with an increased survival rate during the Omicron period, given the understanding that the risk of severe outcomes following SARS-CoV-2 infection is markedly lower for Omicron than for other VOCs. Additionally, the predictors influencing survival may exhibit variations across different VOC periods.

## MATERIALS AND METHODS

This study is a part of a project entitled “SCIFI-PEARL” (Swedish COVID-19 Investigation for Future Insights—a Population Epidemiology Approach using Register Linkage) has received ethical approval from the Swedish Ethical Review Authority (2020-01800 with subsequent amendments—institutional review board statement). This study supports and actively promotes the Declaration of Helsinki principles. We also embrace the 1997 European Convention on Human Rights and Biomedicine (the Oviedo Convention) and the European Union’s General Data Protection Regulation 2016/679. The study follows the Strengthening the Reporting of Observational Studies in Epidemiology guidelines.

### Study Population and Data Sources

This prospective cohort study used data from the SCIFI-PEARL project, a nationwide multilinkage register study of COVID-19 (23). ICU patient data (diagnoses, treatments, and inpatient care/procedures during ICU stay) were mainly obtained from the Swedish Intensive Care Register (SIR). These data were linked with other data sources, including the longitudinal integrated database for health insurance and labor market studies (24) for demographic and socioeconomic factors, and the National Patient Register for prior comorbidities. We identified all adult patients 18 years or older admitted to the ICU for COVID-19 as primary or secondary diagnoses (*International Classification of Diseases*, 10th Edition [ICD-10] U07.1 or U07.2) between March 6, 2020, and September 30, 2022. Episodes of ICU admissions were merged when the gap between discharge and the next admission to an ICU was 2 days or less. We excluded 361 patients (3.9%) with missing data, resulting in 8975 patients for the analysis (**eFig. 1**, http://links.lww.com/CCM/H524). The index date was the date of the first ICU admission. The study population was stratified according to dominant VOC periods in Sweden: pre-Alpha (before January 31, 2021), Alpha (February 1, 2021 to June 30, 2021), Delta (July 1, 2021 to December 31, 2021), and Omicron period (January 1, 2022 to September 19, 2022).

## OUTCOMES

COVID-19-related death in the ICU or after discharge from the ICU was the outcome of interest, identified from the Cause of Death Register (ICD-10 U07.1 or U07.2 as underlying or contributing cause of death). Follow-up extended from the index date to the first: outcome (COVID-19-related death), emigration date, death unrelated to COVID-19, or end of follow-up (December 31, 2022).

## EXPOSURE

The selection of exposure variables was based on existing literature studies, especially baseline demographics, prior comorbidities, and administrative ICU admissions.

1. ICU administrative information: date of ICU admission, arrival route (categories: from a hospital ward, from emergency, from other units [another ICU, intermediate care, other hospitals, maternity ward, etc.]), and type of ICU (general ICU; infectious ICU; and specialized ICUs—neurointensive care, and thoracic intensive care). For treatment strategy, this is determined within 24 hours of care initiation and must be recorded in the patient’s medical record (25) (categories: without restriction, with agreed restrictions in a worsening condition such as omitting or interrupting life-sustaining measures, missing/no decision made).
2. Prior comorbidities from the Swedish Intensive Care Registry for Influenza and Viral Infections module (SIRI) (26) (heart, lung, renal, liver chronic diseases, impaired immune disease, diabetes, morbid obesity (body mass index [BMI] ≥ 40 kg/m^2^), neuromuscular disease, hypertension, and others).
3. Medications received during ICU stay (tocilizumab, remdesivir, baricitinib, steroids, chloroquine phosphate, lopinavir/ritonavir, and others) from the SIRI module (26).
4. Oxygen support interventions/procedures during ICU stay (high-flow nasal cannula (HFNC), continuous positive airway pressure (CPAP), noninvasive ventilation (NIV), invasive mechanical ventilation [IMV]), based on the Swedish medical procedures code (**eTable 1**, http://links.lww.com/CCM/H524). Patients were classified according to the most invasive procedures: 1) no such intervention (2), 2) only HFNC and/or CPAP (3), 3) only NIV, or NIV combined with HFNC or/and CPAP, and 4) only IMV, or IMV combined with NIV/HFNC/CPAP.
5. Organ failures reported during ICU stay, including CNS (e.g., encephalitis), cardiac (e.g., myocarditis), gastrointestinal (e.g., colitis with bloody diarrhea), renal (renal injury unrelated to acute tubular injury associated with circulatory failure/hypoxia), and other.
6. Conditions indicating the severity of the patient’s condition: COVID-19-related acute respiratory distress syndrome and septic shock (eTable 1, http://links.lww.com/CCM/H524).

A list of additional prior comorbidities (eTable 1, http://links.lww.com/CCM/H524) including: stroke, depression, psychiatric disease, and cancer from the National Patient Register (2015–2019).

Sociodemographic and socioeconomic information from Statistic Sweden comprised sex, age at ICU admission (18–39, 40–49, 50–59, 60–69, 70–79, 80+ yr), marital status (married/registered partner, unmarried, divorced/widowed), employment status (full/part-time employed, full/part unemployed, pensioner), education level (primary school, high school, university/college), disposable income (in tertiles representing high, medium, and low-income groups), country of birth (Swedish-born, foreign-born), and healthcare region (Northern, Uppsala-Örebro, Stockholm, South-east, Southern, Western regions) (27).

Vaccination status from the National Vaccination Register. Relevant vaccination status was determined 2 weeks before ICU admission. The patients were categorized as (1) unvaccinated if they had no vaccination before being admitted to ICU or had been vaccinated less than 2 weeks before ICU admission (2), dose 1 (3), dose 2, or (4) three or more doses (booster) if they had the corresponding last dose more than or equal to 2 wk before admission.

### Statistical Analysis

Descriptive analyses included frequencies and percentages for categorical variables and medians and interquartile ranges for continuous variables. Kaplan-Meier curves illustrate estimated survival probabilities, and incidence rates of death were calculated per 1000 person-months. Cox proportional hazard regression, using four models, examined associations between potential covariates and mortality in the entire cohort and separately by VOC periods. Model 0 was unadjusted; model 1 adjusted for baseline covariates (sociodemographics, comorbidities, and ICU admission characteristics); model 2 included treatment strategy, conditions indicating severity, interventions in the ICU, organ failures, and adjusted for baseline covariates; and model 3 included medications during ICU stay, adjusted for baseline covariates. Results are presented as adjusted hazard ratios (aHRs) with 95% CIs. Sensitivity analyses were conducted restricted to patients with a primary diagnosis of COVID-19 for their ICU admission, as well as for age stratification. Because there are few deaths in the young age groups (18–39 and 40–49), these were combined (18–49 yr). All analyses were done with Stata 17.

## RESULTS

### Critically Ill COVID-19 Patient Characteristics

In the entire cohort (**eTable 2**, http://links.lww.com/CCM/H524), 69.5% were men, and the median age was 64 years (interquartile range [IQR] 54–73). Most patients arrived from hospital wards (67.3%) and about one-fourth from the emergency department. The most common comorbidities were hypertension (45%) and diabetes (25.9%). Seven hundred sixty-six (8.5%) patients had morbid obesity (BMI over 40 kg/m^2^), with the highest proportion recorded in the Alpha period (12%). In comparison with other VOC periods, similar characteristics were observed in patients of the Omicron period.

A total of 2927 (32.6%) patients (2080 men and 847 women) died during follow-up (**eTable 3**, http://links.lww.com/CCM/H524), with a mean (sd) age of death of 70 years (10.6) (data not shown). As compared with survivors, the deceased were older (median age 72 vs. 61 yr), retired, divorced/separated/widowed, of lower educational level, vaccinated with only one or two doses, and had chronic conditions such as chronic heart disease, chronic lung disease, impaired immune disease, chronic liver disease, chronic renal disease, diabetes, hypertension, stroke, and cancer.

### Observed Mortality Rates and Temporal Trends

The overall incidence rate of death after ICU care was 19.9 per 1000 person-months (95% CI, 19.2–20.7), with the highest rates observed in the Omicron period, in men and the oldest patients (70+ yr) (**Table 1**). COVID-19 ICU survival rates were higher among younger patients, as demonstrated by the Kaplan-Meier curves in **eFigure 2** (http://links.lww.com/CCM/H524). During the Omicron period, the unadjusted survival probability for those over 70 improved, whereas it declined in the younger age groups. After adjustments, however, patients admitted during the Omicron period were at greater hazard of death (aHR = 1.25; 95% CI, 1.08–1.46) than in the pre-Alpha period (**eTable 4**, http://links.lww.com/CCM/H524). In contrast, the hazard of death in the Alpha and Delta periods was similar to pre-Alpha (aHR = 0.92; 95% CI, 0.84–1.01 and aHR = 1.00; 95% CI, 0.84–1.20).

**TABLE 1.** Crude Incidence Rates of Death Among Adult COVID-19 ICU Patients in Sweden Between March 6, 2020, and September 30, 2022, Overall and by Variant of Concern Periods, Sex, and Age Group

Variables	Person-Months	Deaths	Rate per 1,000 Person-Months	95% CI
Overall	146,894.1	2,927	19.9	19.2–20.7
Variant of concern periods				
Pre-Alpha	92,916.9	1,536	16.5	15.7–17.3
Alpha	40,652.4	765	18.8	17.5–20.2
Delta	6,393.2	169	26.4	22.7–30.7
Omicron	6,931.7	457	65.9	60.2–72.2
Sex				
Women	44,819.6	847	18.9	17.7–20.2
Men	102,074.5	2,080	20.3	19.5–21.3
Age group				
18–39	13,728.8	39	2.8	2.1–.3.9
40–49	18,608.8	87	4.7	3.8–5.7
50–59	60,285.6	645	10.7	9.9–11.6
60–69	20,111.7	480	23.9	21.8–26.1
70+	34,159.2	1,676	49.1	46.8–51.4

### Baseline Sociodemographics, Prior Comorbidities, and ICU Admission Characteristics Associated With Mortality

For the entire cohort, age was the strongest risk for mortality from model 1 (**Table 2**), with HRs ranging from 1.89 (95% CI, 1.29–2.79) up to 11.8 (95% CI, 8.29–16.7). Being male, divorced/separated, having lower income, being from three larger healthcare regions (Stockholm, Southern and Western), and being vaccinated once or twice, were independently associated with mortality. Comorbidities including chronic heart disease, chronic lung disease, impaired immune disease, chronic liver disease, chronic renal disease, stroke, and cancer, were associated with higher mortality (**Table 3**). Furthermore, patients treated in a general ICU had higher mortality than those from a specialized ICU (aHR 1.45; 95% CI, 1.16–1.81). Interestingly, being foreign-born versus Swedish-born (aHR 0.87; 95% CI, 0.80–0.95), and more expectedly having received booster COVID-19 vaccine (aHR 0.69; 95% CI, 0.61–0.79), were associated with better survival (Table 2).

**TABLE 2.** Adjusted Hazard Ratios for the Association Between Baseline Sociodemographics, Vaccination Status, and Mortality (Model 1) Among COVID-19 Patients Admitted to the ICU in Sweden Between March 6, 2020, and September 30, 2022, in the Entire Cohort and Across Variant of Concern Periods

Variables	Pre-Alpha	Alpha	Delta	Omicron	Entire Cohort
	aHR (95% CI)				
Sociodemographic					
Gender (reference women)					
Men	1.12^a^ (1.00–1.26)	1.15^a^ (0.98–1.36)	1.02 (0.70–1.49)	1.09 (0.89–1.35)	1.12^b^ (1.03–1.22)
Age group at ICU admission (reference 18–38 yr)					
40–49	2.18^b^ (1.21–3.94)	1.66 (0.69–3.96)	3.49 (0.72–17.0)	1.85 (0.88–3.91)	1.89^b^ (1.29–2.79)
50–59	4.07^b^ (2.41–6.87)	5.63^b^ (2.62–12.1)	8.41^b^ (1.96–36.1)	2.70^b^ (1.47–4.95)	4.14^b^ (2.95–5.80)
60–69	7.25^b^ (4.22–12.4)	10.61^b^ (4.85–23.18)	38.1^b^ (8.19–177.6)	3.97^b^ (2.05–7.65)	7.42^b^ (5.23–10.5)
70+	12.32^b^ (7.16–21.2)	16.07^b^ (7.32–35.3)	48.8^b^ (10.6–224.2)	5.60^b^ (2.87–10.9)	11.8^b^ (8.29–16.7)
Education level (reference high education)					
Medium education	1.02 (0.89–1.16)	0.99 (0.81–1.19)	0.79 (0.51–1.22)	1.07 (0.82–1.40)	1.00 (0.91–1.10)
Low education	1.09 (0.95–1.26)	1.08 (0.88–1.32)	0.96 (0.61–1.49)	1.06 (0.81–1.40)	1.07 (0.97–1.19)
Marital status (reference married/cohabiting)					
Unmarried	1.11 (0.95–1.30)	1.04 (0.84–1.29)	0.79 (0.46–1.35)	0.93 (0.72–1.21)	1.07 (0.96–1.19)
Divorced/separate	1.10 (0.97–1.24)	1.13 (0.95–1.35)	1.57^c^ (1.07–2.30)	0.91 (0.72–1.15)	1.10^c^ (1.01–1.20)
Income group (reference high income)					
Medium income	1.18^c^ (1.03–1.36)	1.16 (0.96–1.41)	1.08 (0.66–1.75)	1.04 (0.80–1.36)	1.15^b^ (1.04–1.27)
Low income	1.39^b^ (1.19–1.63)	1.09 (0.87–1.36)	1.53 (0.89–2.62)	1.17 (0.87–1.57)	1.28^b^ (1.14–1.44)
Employment (reference employed)					
Unemployed	1.18 (0.92–1.51)	1.18 (0.85–1.64)	1.21 (0.65–2.26)	0.72 (0.44–1.18)	1.15 (0.97–1.37)
Retired	1.18^a^ (0.99–1.40)	0.98 (0.78–1.25)	0.71 (0.40–1.27)	1.06 (0.75–1.51)	1.12^a^ (0.98–1.26)
Country of birth (reference Swedish-born)					
Foreign-born	0.85^b^ (0.75–0.96)	1.03 (0.86–1.23)	0.64^c^ (0.43–0.95)	0.70^b^ (0.53–0.91)	0.87^b^ (0.80–0.95)
Healthcare region (reference Northern region)					
Uppsala/Orebrö	0.97 (0.77–1.22)	0.94 (0.70–1.25)	1.63 (0.68–3.93)	1.03(0.71–1.49)	0.96 (0.82–1.12)
Stockholm	1.28^c^ (1.02–1.59)	1.70^b^ (1.28–2.25)	2.18^a^ (0.94–5.04)	1.64^b^ (1.14–2.37)	1.40^b^ (1.21–1.63)
Southeast	0.72^c^ (0.55–0.94)	0.84 (0.60–1.19)	0.94 (0.34–2.61)	1.05 (0.68–1.62)	0.79^c^ (0.66–0.96)
Southern	1.40^b^ (1.10–1.78)	1.59^b^ (1.17–2.18)	3.62^b^ (1.54–8.52)	1.69^b^ (1.16–2.47)	1.54^b^ (1.31–1.81)
Western	1.21 (0.95–1.53)	1.47^c^ (1.08–2.00)	2.43^a^ (0.97–6.12)	1.52^c^ (1.01–2.27)	1.33^b^ (1.13–1.56)
Vaccination status before ICU admission (reference unvaccinated)					
One dose	7.03^b^ (2.23–22.18)	4.08^b^ (3.11–5.36)	2.41 (0.69–8.37)	1.39 (0.66–2.95)	2.93^b^ (2.33–3.69)
Two doses		3.31^b^ (2.05–5.35)	2.59^b^ (1.71–3.93)	1.70^b^ (1.22–2.36)	2.27^b^ (1.91–2.70)
Three or more doses		0.13^b^ (0.06–0.26)	0.26^b^ (0.14–0.47)	0.80^a^ (0.62–1.03)	0.74^b^ (0.65–0.84)

a *p* < 0.05.

b *p* < 0.001.

c *p* < 0.01.

Note these five regression model results come from the same regressions as in Table 3.

**TABLE 3.** Adjusted Hazard Ratios for the Association Between Prior Comorbidities and Baseline ICU Characteristics and Mortality (Model 1) Among COVID-19 Patients Admitted to the ICU in Sweden Between March 6, 2020, and September 30, 2022, in the Entire Cohort and Across Variant of Concern Periods

Variables	Pre-Alpha	Alpha	Delta	Omicron	Entire Cohort
	aHR (95% CI)				
Prior comorbidities					
Chronic heart diseases^a^	1.33^c^ (1.18–1.51)	1.29^c^ (1.08–1.54)	1.02 (0.66–1.58)	1.17 (0.94–1.45)	1.27^c^ (1.16–1.38)
Chronic lung diseases^a^	1.23^c^ (1.08–1.39)	1.26^c^ (1.06–1.49)	2.48^c^ (1.63–3.77)	1.00 (0.79–1.27)	1.23^c^ (1.12–1.35)
Impaired immune system^a^	1.44^c^ (1.23–1.70)	1.50^c^ (1.20–1.88)	1.47 (0.93–2.34)	1.63^c^ (1.29–2.05)	1.52^c^ (1.36–1.69)
Chronic liver disease^a^	1.45^d^ (0.99–2.12)	2.16^c^ (1.27–3.70)	2.24^d^ (0.96–5.24)	1.21 (0.71–2.08)	1.56^c^ (1.22–2.00)
Chronic renal disease^a^	1.36^c^ (1.15–1.61)	1.36^e^ (1.05–1.76)	3.60^c^ (1.98–6.55)	1.46^c^ (1.12–1.92)	1.45^c^ (1.28–1.63)
Diabetes^a^	1.11^d^ (0.99–1.25)	1.15 (0.97–1.35)	0.76 (0.50–1.16)	0.98 (0.78–1.23)	1.08^d^ (0.99–1.18)
Morbid obesity^a^	0.97 (0.78–1.21)	0.96 (0.74–1.25)	1.49 (0.79–2.82)	1.46^d^ (0.95–2.24)	1.02 (0.88–1.19)
Neuromuscular diseases^a^	1.34^d^ (0.95–1.90)	1.10 (0.49–2.49)	2.26 (0.75–6.83)	1.03 (0.71–1.50)	1.22^d^ (0.97–1.54)
Hypertension^a^	0.93 (0.83–1.03)	0.94 (0.80–1.10)	0.72 (0.49–1.06)	0.86 (0.70–1.06)	0.91^e^ (0.84–0.99)
Other^a^	0.92 (0.81–1.06)	0.92 (0.76–1.11)	0.70 (0.45–1.09)	0.80 (0.60–1.07)	0.91^d^ (0.82–1.00)
Stroke^b^	1.44^c^ (1.11–1.86)	1.54^e^ (1.04–2.27)	1.67 (0.71–3.93)	1.89^c^ (1.23–2.92)	1.57^c^ (1.31–1.89)
Depression^b^	1.08 (0.70–1.67)	1.38 (0.74–2.56)	1.24 (0.12–12.87)	2.17^e^ (1.04–4.50)	1.26 (0.92–1.71)
Psychiatric disease^b^	1.13 (0.77–1.64)	1.01 (0.61–1.68)	0.56 (0.07–4.34)	0.52^e^ (0.28–0.97)	1.02 (0.78–1.33)
Cancer^b^	1.25^c^ (1.08–1.44)	1.19 (0.96–1.47)	1.34 (0.76–2.36)	1.19 (0.93–1.53)	1.25^c^ (1.13–1.39)
ICU admission					
Type of ICU unit (reference specialized ICU)					
General ICU	1.30^d^ (0.96–1.77)	1.35 (0.88–2.06)	1.87 (0.73–4.84)	1.97^e^ (1.09–3.55)	1.45^c^ (1.16–1.81)
Infectious ICU	0.96 (0.67–1.38)	0.81 (0.47–1.37)	2.07 (0.59–7.25)	2.57^e^ (1.11–5.93)	1.08 (0.82–1.41)
Arrival route (reference hospital ward)					
Emergency department	1.08 (0.96–1.22)	1.06 (0.89–1.27)	1.08 (0.72–1.60)	0.73^c^ (0.59–0.91)	1.02 (0.94–1.12)
Other units	1.04 (0.80–1.36)	1.10 (0.83–1.46)	1.10 (0.60–2.01)	0.79 (0.59–1.06)	1.06 (0.91–1.23)

a From Swedish Intensive Care Register data.

b From National Patient Register data.

c *p* < 0.001.

d *p* < 0.05.

e *p* < 0.01.

Note these five regression model results come from the same regressions as in Table 2.

Across VOC periods (Tables 2 and 3), older age, larger healthcare region, prior stroke, chronic renal disease, impaired immune system, and chronic liver disease were associated with higher hazards of death compared with the reference group. The type of ICU unit was only significantly associated with the hazard of death in the Omicron period (general ICU—aHR 1.97; 95% CI, 1.09–3.55, and infectious ICU—aHR 2.57; 95% CI, 1.11–5.93, vs. specialized ICU). ICU patients admitted from emergency departments and “other units” had 27% and 21% lower hazards than those who arrived from hospital wards. The pre-Alpha period demonstrated a higher hazard of death for male ICU patients and patients from lower-income groups or with cancer or diabetes.

### Intervention/Procedures Given During ICU Stay Associated With Mortality

Overall, only 12.8% of COVID-19 ICU patients did not receive any intervention or procedure. 12.5% of ICU patients received only HFNC and/or CPAP, with a higher proportion (14.5%) in the Delta period (**eTable 5**, http://links.lww.com/CCM/H524). Over half of ICU patients required IMV during their stay. The combination of interventions/procedures for ICU patients during different periods (**eFig. 3** and eTable 5, http://links.lww.com/CCM/H524) varied substantially. For example, there was a clear increase in noninvasive respiratory support as the pandemic progressed (13.6–22.3%). The use of IMV decreased across VOC periods. During the first three VOC periods, the proportion of ICU patients not requiring interventions decreased but increased during Omicron. After adjusting for sociodemographics, prior comorbidity, and ICU admission characteristics (model 2), hazards of death among ICU patients in the entire cohort were higher for those with restricted treatment strategy, septic shock, those who received NIV or IMV and for ICU patients with cardiac and other organ failures (*p* < 0.05) (**Table 4**). Across VOC periods, ICU patients with restricted treatment and who required IMV had consistently increased hazards of death.

**TABLE 4.** Adjusted Hazard Ratios for the Association Between Treatment Strategy, Severity Conditions, Interventions/Procedures, Organ Failure During ICU Stay (Model 2), Medications Administered During ICU Stay (Model 3), and Mortality Among ICU Patients in Sweden, in Entire Cohort and by Variant of Concern Periods

Variables	Pre-Alpha	Alpha	Delta	Omicron	Entire cohort
	Adjusted Hazard Ratio (95% CI)				
Treatment strategy, severity conditions, interventions/procedures, organ failure (model 2)					
Treatment strategy (reference without restriction)					
With restriction	6.62^a^ (5.84–7.51)	8.10^a^ (6.77–9.71)	8.67^a^ (5.61–13.4)	7.22^a^ (5.61–9.28)	6.97^a^ (6.36–7.63)
Missing decision	1.11 (0.84–1.48)	1.23 (0.83–1.83)	1.35 (0.62–2.95)	1.32 (0.83–2.10)	1.19^c^ (0.98–1.45)
Severity conditions					
Acute respiratory distress syndrome	0.93 (0.81–1.06)	0.96 (0.79–1.17)	0.75 (0.47–1.20)	0.78^b^ (0.61–1.00)	0.90^b^ (0.82–0.99)
Septic shock	1.33^a^ (1.12–1.59)	1.45^a^ (1.12–1.87)	0.84 (0.42–1.68)	1.46^b^ (1.07–2.00)	1.33^a^ (1.18–1.51)
Oxygen support intervention/procedures during ICU stay (combination)—reference without interventions					
Only HFNC and/or CPAP	0.87 (0.66–1.15)	0.65^c^ (0.42–1.00)	0.57 (0.21–1.55)	1.12 (0.71–1.75)	0.82^b^ (0.67–0.99)
Only NIV or NIV combined with FNC/CPAP	1.37^a^ (1.09–1.72)	1.06 (0.73–1.55)	1.72 (0.80–3.70)	1.13 (0.81–1.58)	1.20^b^ (1.02–1.41)
Only IMV or IMV combined with NIV/HFNC/CPAP	1.39^a^ (1.14–1.71)	1.48^b^ (1.04–2.10)	2.38^b^ (1.14–4.96)	2.05^a^ (1.52–2.76)	1.49^a^ (1.29–1.72)
Organ failure during ICU stay					
CNS	1.24 (0.95–1.63)	0.93 (0.60–1.44)	0.45 (0.08–2.61)	0.97 (0.61–1.54)	1.13 (0.93–1.38)
Cardiac	1.37^a^ (1.08–1.74)	1.01 (0.68–1.50)	5.26^a^ (2.04–13.51)	0.94 (0.58–1.53)	1.21^b^ (1.01–1.45)
Gastrointestinal	1.16 (0.87–1.54)	1.07 (0.58–1.98)	1.20 (0.26–5.47)	1.41 (0.74–2.67)	1.09 (0.87–1.38)
Renal	1.11 (0.93–1.33)	1.17 (0.83–1.63)	0.52 (0.18–1.51)	0.85 (0.52–1.41)	1.05 (0.91–1.21)
Other organs	1.51^a^ (1.27–1.81)	1.37^b^ (1.03–1.82)	1.15 (0.57–2.33)	1.22 (0.91–1.64)	1.34^a^ (1.18–1.52)
Medication received during ICU stay (model 3)					
Chloroquine phosphate	1.35^a^ (1.10–1.66)	—	—	—	1.30^b^ (1.06–1.59)
Tocilizumab	0.96 (0.63–1.47)	0.67^a^ (0.52–0.86)	0.93 (0.61–1.44)	1.20 (0.74–1.93)	0.81^b^ (0.68–0.96)
Lopinavir/ritonavir	0.00 (0.00–0.00)	—	0.00 (0.00–0.00)	10.3^b^ (1.37–77.6)	1.28 (0.18–9.20)
Remdesivir	0.86 (0.71–1.04)	0.97 (0.77–1.22)	0.79 (0.43–1.44)	0.98 (0.74–1.31)	0.98 (0.87–1.11)
Baricitinib	—	7.98^a^ (1.92–33.2)	0.27^c^ (0.07–1.07)	1.01 (0.59–1.73)	1.24 (0.80–1.91)
Steroids	0.95 (0.85–1.06)	0.73^a^ (0.60–0.90)	1.02 (0.64–1.63)	0.83^c^ (0.67–1.03)	0.91^b^ (0.84–0.98)
Other drugs	0.98 (0.82–1.16)	0.95 (0.78–1.16)	0.51^b^ (0.28–0.92)	1.09 (0.81–1.45)	0.99(0.88–1.10)

CPAP = continuous positive airway pressure, HFNC = high-flow nasal cannula, IMV = invasive mechanical ventilation, NIV = noninvasive mechanical ventilation.

a *p* < 0.001.

b *p* < 0.01.

c *p* < 0.05.

### Medications Administered to COVID-19 ICU Patients Associated With Mortality

In the entire cohort, steroids were the most prescribed medication administered to ICU patients (58.7%), followed by remdesivir (10.5%) and tocilizumab (7.1%) (**eTable 6**, http://links.lww.com/CCM/H524). Significant changes in administered medication for COVID-19 ICU patients were observed over the study period. Notably, tocilizumab use increased from 1.7% in the pre-Alpha to 25.3% in the Delta period and decreased to 4.5% in the Omicron period. Remdesivir increased from 8.7% to 13.6% over time. Chloroquine phosphate was only administered in the first VOC period (6.5%). Baricitinib use commenced in the Alpha period and continued until the Omicron period. Trends in medication combinations varied substantially across VOC periods (**eFig. 4**, http://links.lww.com/CCM/H524). During the Omicron period, most ICU patients did not receive any medication. Combined steroids and other drugs (remdesivir, tocilizumab, etc.) increased over the first three VOC periods and decreased in the Omicron period.

Steroids lowered death risk (aHR 0.91; 95% CI, 0.84–0.98) (Table 4), and this effect was stronger and remained significant only in the Alpha period. Chloroquine phosphate was associated with a higher hazard of 1.35 (95% CI, 1.10–1.66) in the pre-Alpha period when it was used. Patients with lopinavir/ritonavir administered during the Omicron period had a 10.3 times higher hazard of death (95% CI, 1.37–77.6).

### Sensitivity Analysis

The effect estimates from the sensitivity analysis, which included only ICU patients with COVID-19 as the primary diagnosis (*n* = 6533), were similar to those in the main analysis (**eTables 7** and **8**, http://links.lww.com/CCM/H524). According to sensitivity analyses across age groups (**eTables 9** and **10**, http://links.lww.com/CCM/H524), there are relatively consistent risk factors with significantly higher mortality hazards as in the main findings of the study.

## DISCUSSION

In this Swedish prospective population-based cohort of COVID-19, ICU patients studied over the initial two and a half years of the pandemic, the survival rate among COVID-19 critically ill patients appears to have changed over time, with a worse survival in the Omicron period overall. The aHR comparing older and younger ages was consistently strong but slightly attenuated in the Omicron period. Several predictors of increased mortality risk were identified across VOC periods, including older age, prior chronic heart disease, chronic lung disease, impaired immune system, chronic liver disease, chronic renal disease, stroke, cancer, ICU patients with restricted treatment, requiring IMV and receiving chloroquine phosphate. Conversely, foreign-born origin, booster vaccination, and steroid administration significantly reduced the risk of mortality.

The observed variation in survival rate over time aligns with findings in previous studies (13, 28–31). Possible reasons for such temporal variation include variations in follow-up, particularly with the Omicron period having shorter follow-up, but changes inpatient characteristics, such as underlying medical conditions and needs of IMV, along with improvements in clinical management, could also contribute. Despite the Omicron variant generally being associated with a milder clinical course, we found the mortality risk was somewhat higher in ICU patients infected during the Omicron period compared with other VOC periods; and older groups had a higher mortality risk than the youngest group, even though this age difference was less pronounced during Omicron period. The characteristics of COVID-19 ICU patients during the Omicron period may explain this—they were older and retired, had a higher prevalence of prior comorbidities (such as chronic heart disease, impaired immunity, chronic liver disease, neuromuscular disease, psychiatric disease, and cancer), had a low income, developed a severe condition (septic shock) and organ failures. Nevertheless, as compared with the first three periods, ICU survival in the elderly improved in the Omicron period both in the unadjusted and adjusted analyses. It appears that vaccination status plays an important role, as the aHR for death in the Omicron period for the elderly falls to 0.74 (point estimate) for booster vaccine.

We identified significant mortality predictors across different VOC periods after adjusting for sociodemographics, comorbidities, and ICU admission characteristics. The hazards of death were higher among ICU patients who were older, had impaired immune system disease, and with restricted treatment strategies. Our findings align with a previous Swedish study during the early pandemic (19). We also found that men constituted a disproportionate fraction of critical care cases, consistent with prior meta-analysis evidence (12). Age is a well-known predictor for COVID-19 mortality, particularly for critical care patients (32). In addition, ICU patients are generally sicker at the time of their admission. Elderly patients are at risk of developing severe COVID-19 and exhibiting increased mortality (33), indicating age remains a strong independent risk factor for mortality regardless of variants, as confirmed in this study. As found in other European studies, foreign-born individuals had a lower mortality risk (34, 35). Prior comorbidities consistently predict mortality across different periods, aligning with earlier studies (10, 13, 21).

Initially, during the pandemic, medications like chloroquine phosphate were tried but then halted due to safety concerns, including heart rhythm issues, blood and lymph system disorders, kidney injury, and liver failure (36, 37). Our results show a 44% increase in the hazard of death among adult Swedish COVID-19 ICU patients who received chloroquine phosphate. Previous studies support the early use of dexamethasone early in the progression of COVID-19 disease (38, 39). Steroids, the most prescribed ICU medication in Sweden, significantly reduced mortality risk over time, consistent with prior studies (40–42). We found an increased risk of death for patients who received baricitinib, contrary to other results (30, 43–45), possibly indicating reverse causality, with baricitinib treatment being an indicator of high-risk patients. This study should be interpreted cautiously as baricitinib was first administered during the Alpha variant period in Sweden, involving only four patients.

As anticipated, ICU patients requiring NIV or IMV, and with organ failures and severity conditions, had a higher hazard of death, consistent with earlier studies (46, 47). Given the diverse underlying medical conditions, varying illness severity levels, and the need for IMV, analyzing and interpreting the impact of VOC periods on mortality during the pandemic in Sweden poses a challenge.

We found that, upon admission, having previously received one or two vaccinations was insufficient to prevent mortality in ICU patients with severe medical conditions. Possible reasons include that the oldest and highest-risk persons were targeted first with doses 1–2 when vaccine availability was still limited, and that vaccination closely before ICU admission did not yet confer adequate protection. Our study consistently demonstrated that receiving booster vaccines before ICU admission was a protective factor against death across VOC periods, aligning with prior evidence (48).

This study’s extensive database combining data from the SIR with data from national healthcare databases allows a comprehensive and detailed view of ICU patient history at an individual level. The database is updated every 3 months, supporting the inclusion of both prospective and recent register data. Encompassing over two and a half years of the pandemic, the study covers various VOC periods, offering a detailed insight into critical care treatment of COVID-19 ICU patients in Sweden, including the use of remdesivir, steroids, anticoagulation, tocilizumab, and last but not least, vaccination for COVID-19 (1). However, some limitations must also be considered. Comparing exposures across various VOC periods in critical care, as done here, is often confounded by variations in disease severity among patients and treatment given (confounding by indication). Like all observational studies, the results of this study do not necessarily indicate a causal relationship. Some unmeasured confounders may affect the estimates, such as health risk behavior (physical activity, smoking), that are unavailable in the database.

## CONCLUSIONS

In this nationwide Swedish cohort covering two and a half years of the pandemic, we observed that ICU survival rates changed over time. Older age was a strong predictor across all VOC periods. Furthermore, most other mortality predictors remained consistent across different variant periods after adjustment for potential confounders. The study presents valuable information to clinicians about the evolving procedures, treatments, clinical conditions, and outcomes of COVID-19 ICU patients, which is important for potential COVID-19 resurgence and future pandemic planning.

## ACKNOWLEDGMENTS

Swedish COVID-19 Investigation for Future Insights—a Population Epidemiology Approach using Register Linkage team members provided insight, assistance, and guidance, greatly benefiting the research. The authors thank the Swedish register holders: Statistics Sweden, Social Styrelsen, and the Public Health Agency of Sweden for providing us with register data. Further acknowledgments are due to all patients and healthcare professionals who contributed to Swedish Intensive Care Register (SIR) registrations, the SIR steering committee, and register coordinators.

## Supplementary Material


