# Supplementary material for: Survival of Critically Ill COVID-19 Patients in Sweden During the First Two and a Half Years of the Pandemic*
Source: Crit Care Med. 2024 Mar 28;52(8):1194–205. doi: 10.1097/CCM.0000000000006271 (PMC11239088; doi:10.1097/CCM.0000000000006271)

Online Supplementary text

Survival of critically ill patients with COVID-19 during two-and-a-half years of the pandemic in Sweden

Ailiana Santosa, Jonatan Oras, Huiqi Li, Chioma Nwaru, Brian Kirui, Fredrik Nyberg

**eTable 1**	ICD-10 codes for prior comorbidities from NPR and severity of the patient's condition and Swedish medical procedures (KVÅ) code for Interventions/treatment procedures from the Swedish Intensive Care Register
**eTable 2**	Baseline demographic, socioeconomic, prior comorbidity, and ICU admission characteristics of adult COVID-19 ICU patients in Sweden between 6 March 2020 and 30 September 2022, overall and by VOC periods, n (%)
**eTable 3**	Baseline demographic, socioeconomic, prior comorbidity, and ICU admission characteristics of adult COVID-19 ICU patients in Sweden between 6 March 2020 and 30 September 2022, by survivorship (n (%))
**eTable 4**	Unadjusted and adjusted hazards of mortality among COVID-19 ICU patients by the variant of concern periods
**eTable 5**	Intervention/procedures, adverse symptoms, and severity characteristics of COVID-19 ICU patients in Sweden between 6 March 2020 and 30 September 2022, in the entire cohort and across variant of concern periods, (n (%)).
**eTable 6**	Medications received during ICU admission of COVID-19 ICU patients in Sweden between 6 March 2020 and 30 September 2022, in the entire cohort and across VOC periods (n (%)).
**eTable 7**	Sensitivity analysis restricting the study cohort only to patients with *primary* diagnosis of COVID-19 admitted to the ICU in the entire cohort for the association between sociodemographic, prior comorbidities, ICU admission characteristics and mortality (model 1) among ICU patients in Sweden between 6 March 2020 and 30 September 2022.
**eTable 8**	Sensitivity analysis restricting the study cohort only to patients with *primary* diagnosis of COVID-19 admitted to the ICU in the entire cohort for the association between treatment strategy, severity conditions, interventions/procedures, organ failure during ICU stay (model 2), medications administered during ICU stay (model 3) and mortality among ICU patients in Sweden.
**eTable 9**	Sensitivity analysis for the association between sociodemographic, prior comorbidities, ICU admission and mortality (model 1) among ICU patients in Sweden between 6 March 2020 and 30 September 2022 in the entire cohort, by age group
**eTable 10**	Sensitivity analysis for the association between treatment strategy, severity conditions, interventions/procedures, organ failure during ICU stay (model 2), medications administered during ICU stay (model 3) and mortality among ICU patients in Sweden in the entire cohort, by age group.
**eFigure 1**	Study flowchart of study participant
**eFigure 2**	Plots of Kaplan-Meier estimates of survival of COVID-19 patients admitted to ICU by variant of concern periods.
**eFigure 3**	COVID-19 intervention/procedures in critical care patients across different variant of concern periods.
**eFigure 4**	COVID-19-specific medication received during ICU stay in critical care patients across variant of concern periods.

eTable 1. ICD-10 codes for prior comorbidities from National Patient Register and severity of the patient's condition and Swedish medical procedures (KVÅ) code for Interventions/treatment procedures from the Swedish Intensive Care Register

Prior comorbidities[¥]	ICD-10 code
Stroke	I60-I62, I630-I635, I638-I639, I65
Depression	F32-F33
Psychiatric disease	F20-F39
Cancer	C00-C97
Severity of the patient's condition during ICU stay[§]	ICD-10 code
Acute Respiratory Distress Syndrome (ARDS)	J809, J809A, J809B, J809C, J809X
Septic shock	R57.2
Interventions/treatment procedures during ICU stay[§]	Medical procedures code (KVÅ)
Invasive Mechanical Ventilation support (IMV)	DG021
Non-invasive mechanical ventilation support (NIV)	DG023
High-flow nasal cannula (HFNC)	DG028
Continuous Positive Airway Pressure (CPAP)	DG001

[§]from Swedish Intensive Care Register data. [¥] from National Patient Register data.

eTable 2. Baseline demographic, socioeconomic, prior comorbidity, and ICU admission characteristics of adult COVID-19 ICU patients in Sweden between 6 March 2020 and 30 September 2022, overall and by VOC periods, n (%)

**Covariates**	**Total (n=8975)**	**Pre-Alpha (n=4618)**	**Alpha (n=2642)**	**Delta (n=601)**	**Omicron (n=1114)**
Sociodemographics					
Sex					
Men	6241 (69.5)	3323 (72.0)	1798 (68.1)	407 (67.7)	713 (64.0)
Women	2734 (30.5)	1295 (28.0)	844 (31.9)	194 (32.3)	401 (36.0)
Age at ICU admission (median, IQR)	64 (54;73)	64 (55;72)	63 (53;72)	60 (47;71)	69 (55;77)
Age group					
18-39	668 (7.4)	277 (6.0)	184 (7.0)	85 (14.1)	122 (11.0)
40-49	886 (9.9)	406 (8.8)	313 (11.8)	88 (14.6)	79 (7.1)
50-59	3058 (34.1)	1682 (36.4)	934 (35.4)	197 (32.8)	245 (22.0)
60-69	1275 (14.2)	692 (15.0)	383 (14.5)	59 (9.8)	141 (12.7)
70+	3088 (34.4)	1561 (33.8)	828 (31.3)	172 (28.6)	527 (47.3)
Marital status					
Married/cohabiting	4629 (51.6)	2546 (55.1)	1379 (52.2)	297 (49.4)	407 (36.5)
Unmarried	2095 (23.3)	955 (20.7)	631 (23.9)	149 (24.8)	360 (32.3)
Divorced/separated/widow	2251 (25.1)	1117 (24.2)	632 (23.9)	155 (25.8)	347 (31.1)
Education					
University/college	2293 (25.5)	1213 (26.3)	675 (25.5)	147 (24.5)	258 (23.2)
High school	4013 (44.7)	2082 (45.1)	1201 (45.5)	261 (43.4)	469 (42.1)
Primary school	2669 (29.7)	1323 (28.6)	766 (29.0)	193 (32.1)	387 (34.7)
Income group					
High income	3071 (34.2)	1679 (36.4)	968 (36.6)	151 (25.1)	273 (24.5)
Medium income	3051 (34.0)	1536 (33.3)	902 (34.1)	213 (35.4)	400 (35.9)
Low income	2853 (31.8)	1403 (30.4)	772 (29.2)	237 (39.4)	441 (39.6)
Employment					
Employed	3964 (44.2)	2109 (45.7)	1267 (48.0)	271 (45.1)	317 (28.5)
Unemployed	860 (9.6)	393 (8.5)	261 (9.9)	104 (17.3)	102 (9.2)
Retired	4151 (46.3)	2116 (45.8)	1114 (42.2)	226 (37.6)	695 (62.4)
Country of birth					
Swedish born	5732 (63.9)	2788 (60.4)	1776 (67.2)	295 (49.1)	873 (78.4)
Foreign born	3243 (36.1)	1830 (39.6)	866 (32.8)	306 (50.9)	241 (21.6)
Healthcare region					
Northern region	750 (8.4)	318 (6.9)	269 (10.2)	44 (7.3)	119 (10.7)
Uppsala/Orebrö	2019 (22.5)	954 (20.7)	678 (25.7)	117 (19.5)	270 (24.2)
Stockholm	2525 (28.1)	1433 (31.0)	631 (23.9)	182 (30.3)	279 (25.0)
Southeast region	937 (10.4)	459 (9.9)	287 (10.9)	65 (10.8)	126 (11.3)
Southern region	1089 (12.1)	556 (12.0)	286 (10.8)	90 (15.0)	157 (14.1)
Western region	1655 (18.4)	898 (19.4)	491 (18.6)	103 (17.1)	163 (14.6)
Vaccination status before ICU admission					
Unvaccinated	7794 (86.8)	4615 (99.9)	2446 (92.6)	417 (69.4)	316 (28.4)
One dose	91 (1.0)	3 (0.1)	66 (2.5)	5 (0.8)	17 (1.5)
Two doses	242 (2.7)	0 (0.0)	23 (0.9)	79 (13.1)	140 (12.6)
Three or more doses	848 (9.4)	0 (0.0)	107 (4.0)	100 (16.6)	641 (57.5)
**Prior comorbidities**					
Chronic heart diseases§	1591 (17.7)	805 (17.4)	416 (15.7)	80 (13.3)	290 (26.0)
Chronic lung diseases§	1615 (18.0)	806 (17.5)	521 (19.7)	82 (13.6)	206 (18.5)
Impaired immune§	893 (9.9)	387 (8.4)	233 (8.8)	69 (11.5)	204 (18.3)

Chronic liver disease§	115 (1.3)	50 (1.1)	25 (0.9)	10 (1.7)	30 (2.7)
Chronic renal disease§	614 (6.8)	307 (6.6)	150 (5.7)	30 (5.0)	127 (11.4)
Diabetes§	2325 (25.9)	1251 (27.1)	641 (24.3)	153 (25.5)	280 (25.1)
Morbid obesity§	766 (8.5)	337 (7.3)	317 (12.0)	51 (8.5)	61 (5.5)
Neuromuscular diseases§	180 (2.0)	81 (1.8)	20 (0.8)	7 (1.2)	72 (6.5)
Hypertension§	4037 (45.0)	2131 (46.1)	1237 (46.8)	228 (37.9)	441 (39.6)
Other§	1458 (16.2)	788 (17.1)	441 (16.7)	90 (15.0)	139 (12.5)
Stroke¥	212 (2.4)	108 (2.3)	54 (2.0)	13 (2.2)	37 (3.3)
Depression¥	397 (4.4)	206 (4.5)	99 (3.7)	21 (3.5)	71 (6.4)
Psychiatric disease¥	591 (6.6)	294 (6.4)	157 (5.9)	29 (4.8)	111 (10.0)
Cancer¥	866 (9.6)	452 (9.8)	234 (8.9)	39 (6.5)	141 (12.7)
**ICU admission§**					
Type of ICU unit					
Specialised ICU	340 (3.8)	176 (3.8)	83 (3.1)	28 (4.6)	53 (4.7)
General ICU	7838 (87.5)	3938 (85.4)	2351 (89.1)	517 (86.2)	1032 (93.4)
Infectious ICU	797 (8.9)	504 (10.9)	208 (7.9)	56 (9.3)	29 (2.6)
Arrival route					
Hospital ward	6036 (67.3)	3295 (71.4)	1888 (71.5)	363 (60.4)	490 (44.0)
Emergency unit	2355 (26.2)	1141 (24.7)	582 (22.0)	169 (28.1)	463 (41.6)
Other units	584 (6.5)	182 (3.9)	172 (6.5)	69 (11.5)	161 (14.5)

Footnote: VOC: Variant of concern. ICU – Intensive Care Unit, §from Swedish Intensive Care Register data. ¥ from National Patient Register data

eTable 3. Baseline demographic, socioeconomic, prior comorbidity, and ICU admission characteristics of adult COVID-19 ICU patients in Sweden between 6 March 2020 and 30 September 2022, by survivorship (n (%))

**Covariates**	**Total (n=8975)**	**Survivor (n=6048)**	**Deceased (n=2927)**
Sociodemographics			
Sex			
Men	6241 (69.5)	4161 (68.8)	2080 (71.1)
Women	2734 (30.5)	1887 (31.2)	847 (28.9)
Age at admission (median, IQR)	64 (54-73)	61 (51;70)	72 (64;77)
Age group			
18-39	668 (7.4)	629 (10.4)	39 (1.3)
40-49	886 (9.9)	799 (13.2)	87 (3.0)
50-59	3058 (34.1)	2413 (39.9)	645 (22.0)
60-69	1275 (14.2)	795 (13.1)	480 (16.4)
70+	3088 (34.4)	1412 (23.3)	1676 (57.3)
Marital status			
Married/cohabiting	4629 (51.6)	3152 (52.1)	1477 (50.5)
Unmarried	2095 (23.3)	1554 (25.7)	541 (18.5)
Divorced/separated/widow	2251 (25.1)	1342 (22.2)	909 (31.1)
Education			
University/college	2293 (25.5)	1616 (26.7)	677 (23.1)
High school	4013 (44.7)	2767 (45.8)	1246 (42.6)
Primary school	2669 (29.7)	1665 (27.5)	1004 (34.3)
Income group			
High income	3071 (34.2)	2258 (37.3)	813 (27.8)
Medium income	3051 (34.0)	1999 (33.1)	1052 (35.9)
Low income	2853 (31.8)	1791 (29.6)	1062 (36.3)

Employment			
Employed	3964 (44.2)	3233 (53.5)	731 (25.0)
Unemployed	860 (9.6)	667 (11.0)	193 (6.6)
Retired	4151 (46.3)	2148 (35.5)	2003 (68.4)
Country of birth			
Swedish born	5732 (63.9)	3717 (61.5)	2015 (68.8)
Foreign born	3243 (36.1)	2331 (38.5)	912 (31.2)
Healthcare region			
Northern region	750 (8.4)	522 (8.6)	228 (7.8)
Uppsala/Orebrö	2019 (22.5)	1488 (24.6)	531 (18.1)
Stockholm	2525 (28.1)	1614 (26.7)	911 (31.1)
Southeast region	937 (10.4)	699 (11.6)	238 (8.1)
Southern region	1089 (12.1)	638 (10.5)	451 (15.4)
Western region	1655 (18.4)	1087 (18.0)	568 (19.4)
Vaccination status before ICU admission			
Unvaccinated	7685 (85.6)	5326 (88.1)	2359 (80.6)
One dose	133 (1.5)	15 (0.2)	118 (4.0)
Two doses	246 (2.7)	99 (1.6)	147 (5.0)
Three or more doses	911 (10.2)	608 (10.1)	303 (10.4)
**Prior comorbidities**			
Chronic heart diseases§	1591 (17.7)	791 (13.1)	800 (27.3)
Chronic lung diseases§	1615 (18.0)	962 (15.9)	653 (22.3)
Impaired immune disease§	893 (9.9)	455 (7.5)	438 (15.0)
Chronic liver disease§	115 (1.3)	46 (0.8)	69 (2.4)
Chronic renal disease§	614 (6.8)	278 (4.6)	336 (11.5)
Diabetes§	2325 (25.9)	1428 (23.6)	897 (30.6)
Morbid obesity§	766 (8.5)	571 (9.4)	195 (6.7)
Neuromuscular diseases§	180 (2.0)	104 (1.7)	76 (2.6)
Hypertension§	4037 (45.0)	2490 (41.2)	1547 (52.9)
Other§	1458 (16.2)	976 (16.1)	482 (16.5)
Stroke¥	212 (2.4)	92 (1.5)	120 (4.1)
Depression¥	397 (4.4)	268 (4.4)	129 (4.4)
Psychiatric disease¥	591 (6.6)	403 (6.7)	188 (6.4)
Cancer¥	866 (9.6)	398 (6.6)	468 (16.0)
**ICU admission§**			
Type of ICU unit			
Specialised ICU	319 (3.6)	230 (3.8)	89 (3.0)
General ICU	7838 (87.5)	5239 (86.8)	2599 (89.0)
Infectious ICU	797 (8.9)	566 (9.4)	231 (7.9)
Arrival route			
Hospital ward	6036 (67.3)	4057 (67.1)	1979 (67.6)
Emergency unit	2355 (26.2)	1606 (26.6)	749 (25.6)
Other units	584 (6.5)	385 (6.4)	199 (6.8)

Footnote: §from Swedish Intensive Care Register data. ¥ from National Patient Register data. VOC: Variant of concern. ICU: Intensive Care Unit.

eTable 4. Unadjusted and adjusted hazards of mortality among COVID-19 ICU patients, by the VOC periods

	Unadjusted HR (95%CI)	p value	Adjusted HR# (95%CI)	p-value
Alpha	0.89 (0.82-0.98)	0.014	0.92 (0.84-1.01	0.078
Delta	0.90 (0.77-1.06)	0.195	1.00 (0.84-1.20)	0.980
Omicron	1.49 (1.34-1.65)	0.000	1.25 (1.08-1.46)	0.04

VOC: Variant of concern; HR: Hazard ratio. Reference group: pre-alpha period

#Adjusted for sociodemographics, prior comorbidities, ICU admission characteristics

eTable 5. Oxygen support intervention/procedures, adverse symptoms, and severity characteristics of COVID-19 ICU patients in Sweden between 6 March 2020 and 30 September 2022, in the entire cohort and across variant of concern periods, (n (%).

**Variables**	**Total (n=8975)**	**Pre-Alpha (n=4618)**	**Alpha (n=2642)**	**Delta (n=601)**	**Omicron (n=1114)**
**Treatment strategy**					
Without restriction	6037 (67.3)	3107 (67.3)	1858 (70.3)	420 (69.9)	652 (58.5)
With restriction	2310 (25.7)	1209 (26.2)	608 (23.0)	131 (21.8)	362 (32.5)
Missing decision	628 (7.0)	302 (6.5)	176 (6.7)	50 (8.3)	100 (9.0)
**Severity conditions**					
ARDS	6185 (68.9)	3447 (74.6)	1972 (74.6)	421 (70.0)	345 (31.0)
Septic shock	535 (6.0)	286 (6.2)	125 (4.7)	25 (4.2)	99 (8.9)
**Oxygen support intervention/procedures during ICU stay (combination)**					
None	1148 (12.8)	554 (12.0)	230 (8.7)	59 (9.8)	305 (27.4)
Only HFNC and/or CPAP	1119 (12.5)	565 (12.2)	371 (14.0)	87 (14.5)	96 (8.6)
Only NIV or NIV combined with FNC/CPAP	1510 (16.8)	630 (13.6)	537 (20.3)	134 (22.3)	209 (18.8)
Only IMV or IMV combined with NIV/HFNC/CPAP	5198 (57.9)	2869 (62.1)	1504 (56.9)	321 (53.4)	504 (45.2)
**Organ failure during ICU stay**					
CNS	245 (2.8)	129 (2.9)	57 (2.2)	11 (1.8)	48 (4.6)
Cardiac	285 (3.2)	159 (3.5)	69 (2.6)	11 (1.8)	46 (4.4)
Gastrointestinal	204 (2.3)	131 (2.9)	37 (1.4)	13 (2.2)	23 (2.2)
Renal	461 (5.2)	308 (6.8)	88 (3.3)	18 (3.0)	47 (4.5)
Other	735 (8.4)	378 (8.4)	167 (6.3)	47 (7.8)	143 (13.6)

Footnote**:** VOC: Variant of concern. ARDS-acute respiratory distress syndrome. HFNC-High-Flow Nasal Cannula. CPAP-Continuous Positive Airway Pressure. NIV-Non-Invasive Mechanical Ventilation. IMV-Invasive Mechanical Ventilation.

CNS: Central Nervous System

eTable 6. Medications received during ICU admission of COVID-19 ICU patients in Sweden between 6 March 2020 and 30 September 2022, in the entire cohort and across VOC periods (n (%))

**Variables**	**Total (n=8975)**	**Pre-Alpha (n=4618)**	**Alpha (n=2642)**	**Delta (n=601)**	**Omicron (n=1114)**
Chloroquin-phosphate	301 (3.4)	301 (6.5)	0 (0.0)	0 (0.0)	0 (0.0)
Tocilizumab	635 (7.1)	78 (1.7)	355 (13.4)	152 (25.3)	50 (4.5)
Lopinavir/Ritonavir	4 (0.0)	2 (0.0)	0 (0.0)	1 (0.2)	1 (0.1)
Remdesivir	940 (10.5)	404 (8.7)	319 (12.1)	65 (10.8)	152 (13.6)
Baricitinib	46 (0.5)	0 (0.0)	3 (0.1)	13 (2.2)	30 (2.7)
Steroids	5264 (58.7)	2092 (45.3)	2232 (84.5)	491 (81.7)	449 (40.3)
Other drug	1143 (12.7)	400 (8.7)	507 (19.2)	97 (16.1)	139 (12.5)

Footnote**:** VOC: Variant of concern. ICU – Intensive Care Unit.

eTable 7. Sensitivity analysis restricting the study cohort only to patients with *primary* diagnosis of COVID-19 patients admitted to the ICU in the entire cohort for the association between sociodemographic, prior comorbidities, ICU admission characteristics and mortality (model 1) among ICU patients in Sweden between 6 March 2020 and 30 September 2022.

**Variables**	**aHR (95%CI)**
**Sociodemographic**	
Men (ref. women)	1.15***(1.04-1.28)
Age group (ref. 18-39 year)	
40-49	1.54(0.91-2.59)
50-59	4.08***(2.59-6.41)
60-69	7.59***(4.77-12.07)
70+	11.94***(7.48-19.1)
Education (ref. high education)	
Medium education	0.98(0.88-1.10)
Low education	1.04(0.93-1.18)
Marital status (ref. married/cohabiting)	
Unmarried	1.14**(1.00-1.30)
Divorced/separate	1.09*(0.99-1.21)
Income group (ref. high income)	
Medium income	1.17***(1.05-1.32)
Low income	1.32***(1.16-1.51)
Employment (ref. employed)	
Unemployed	1.02(0.82-1.26)
Retired	1.11(0.96-1.29)
Country of birth (ref. Swedish-born)	
Foreign-born	0.86***(0.78-0.96)
Healthcare region (ref. Northern region)	
Uppsala/Orebrö	0.89(0.74-1.08)
Stockholm	1.37***(1.16-1.63)
Southeast region	0.70***(0.57-0.86)
Southern region	1.45***(1.20-1.75)
Western region	1.37***(1.13-1.65)
Vaccination status before ICU admission (ref. unvaccinated)	
One dose	2.79***(2.11-3.69)
Two doses	2.71***(2.18-3.36)
Three or more doses	0.64***(0.53-0.78)

Prior comorbidities	
Chronic heart diseases§	1.28***(1.15-1.41)
Chronic lung diseases§	1.27***(1.15-1.42)
Impaired immune system§	1.56***(1.37-1.78)
Chronic liver disease§	1.52**(1.09-2.12)
Chronic renal disease§	1.48***(1.28-1.70)
Diabetes§	1.11**(1.00-1.23)
Morbid obesity§	0.98(0.83-1.17)
Neuromuscular diseases§	1.26(0.91-1.73)
Hypertension§	0.90**(0.82-0.99)
Other§	0.89*(0.79-1.00)
Stroke¥	1.42***(1.12-1.79)
Depression¥	1.34(0.91-1.97)
Psychiatric disease¥	0.99(0.71-1.38)
Cancer¥	1.22***(1.08-1.39)
**ICU Admission characteristics**	
Type of ICU unit (ref. specialised ICU)	
General ICU	1.63***(1.19-2.24)
Infectious ICU	1.15(0.80-1.64)
Arrival route (ref. hospital ward)	
Emergency department	0.97(0.87-1.08)
Other units	1.02(0.83-1.25)

Footnote: aHR – adjusted hazard ratio, ICU – Intensive Care Unit, BMI – Body Mass Index.
§from Swedish Intensive Care Register data. ¥ from National Patient Register data.
*p<0.05, **p<0.01, ***p<0.001

eTable 8. Sensitivity analysis restricting the study cohort only to patients with *primary* diagnosis of COVID-19 admitted to the ICU in the entire cohort for the association between treatment strategy, severity conditions, interventions/procedures, organ failure during ICU stay (model 2), medications administered during ICU stay (model 3) and mortality among ICU patients in Sweden.

	**aHR (95%CI)**
**Model 2**	
Treatment strategy (ref: without restriction)	
With restriction	7.26***(6.53 - 8.08)
Missing decision	1.07(0.84 - 1.38)
Severity conditions	
ARDS	0.99(0.88 - 1.11)
Septic shock	1.32***(1.14 - 1.54)
Oxygen support intervention/procedures during ICU stay (combination) – ref. without interventions	
Only HFNC and/or CPAP	0.73**(0.56 - 0.94)
Only NIV or NIV combined with FNC/CPAP	1.21*(0.97 - 1.50)
Only IMV or IMV combined with NIV/HFNC/CPAP	1.27**(1.03 - 1.56)
Organ failure during ICU stay	
CNS	0.96(0.73 - 1.25)
Cardiac	0.90(0.71 - 1.14)
Gastrointestinal	1.14(0.86 - 1.50)
Renal	1.16*(0.97 - 1.38)
Other organ	1.46***(1.23 - 1.73)
**Model 3**	
Medications administered during ICU stay	
Chloroquin-phosphate	1.28**(1.01 - 1.63)

Tocilizumab	0.78***(0.65 - 0.94)
Lopinavir-Ritonavir	2.37(0.33 - 17.21)
Remdesivir	1.03(0.89 - 1.18)
Baricitinib	1.25(0.77 - 2.03)
Steroids	0.93(0.84 - 1.03)
Other drug	1.00(0.88 - 1.13)

Footnote: VOC: Variant of concern. aHR – adjusted hazard ratio. ARDS-acute respiratory distress syndrome. HFNC-High-Flow Nasal Cannula. CPAP-Continuous Positive Airway Pressure. NIV-Non-Invasive Mechanical Ventilation. IMV-Invasive Mechanical Ventilation. CNS: Central Nervous System

*p<0.05, **p<0.01, ***p<0.001

eTable 9. Sensitivity analysis for the association between sociodemographic, prior comorbidities, ICU admission and mortality (model 1) among ICU patients with primary diagnosis of COVID-19 at admission in Sweden between 6 March 2020 and 30 September 2022 in the entire cohort, *by age group*

**Variables**	**18-49 year**	**50-59 year**	**60-69 year**	**70+**
	**aHR (95%CI)**	**aHR (95%CI)**	**aHR (95%CI)**	**aHR (95%CI)**
Gender (ref. women)				
Men	1.28(0.87-1.88)	1.18*(0.99-1.42)	1.21*(0.97-1.50)	1.08(0.97-1.21)
Marital status (ref. married/cohabiting)				
Unmarried	1.27(0.83-1.94)	0.99(0.81-1.22)	1.09(0.83-1.41)	1.03(0.88-1.21)
Divorced/separate	1.07(0.58-1.97)	1.06(0.87-1.29)	0.94(0.74-1.18)	1.11*(0.99-1.24)
Education level (ref. high education)				
Medium education	0.97(0.63-1.50)	1.03(0.84-1.26)	0.91(0.72-1.15)	1.01(0.88-1.15)
Low education	0.51**(0.29-0.88)	1.11(0.88-1.40)	1.00(0.77-1.29)	1.11(0.97-1.27)
Income group (ref. high income)				
Medium income	1.30(0.79-2.14)	1.25**(1.02-1.54)	1.19(0.94-1.51)	1.13*(0.99-1.30)
Low income	1.46(0.83-2.57)	1.44***(1.12-1.84)	1.31*(1.00-1.72)	1.26***(1.08-1.47)
Employment (ref. employed)				
Unemployed	1.50(0.91-2.49)	0.98(0.76-1.27)	1.01(0.64-1.59)	1.72**(1.06-2.78)
Retired	1.45(0.69-3.02)	1.26*(0.99-1.61)	1.16(0.93-1.45)	0.93(0.75-1.16)
Country of birth (ref. Swedish-born)				
Foreign-born	1.24(0.82-1.86)	0.90(0.74-1.08)	0.87(0.70-1.09)	0.85***(0.75-0.96)
Healthcare region (ref. Northern region)				
Uppsala/Orebrö	1.43(0.54-3.79)	1.17(0.81-1.69)	0.85(0.59-1.23)	0.90(0.73-1.10)
Stockholm	2.94**(1.14-7.53)	1.79***(1.26-2.56)	1.05(0.73-1.52)	1.33***(1.09-1.61)
Southeast	1.37(0.47-3.97)	1.07(0.70-1.64)	0.58**(0.37-0.9)	0.74**(0.58-0.94)
Southern	2.21(0.79-6.15)	1.84***(1.24-2.71)	1.21(0.82-1.79)	1.53***(1.25-1.89)
Western	2.52*(0.91-6.96)	1.52**(1.02-2.28)	1.24(0.85-1.82)	1.27**(1.03-1.56)
Vaccination status before ICU admission (ref. unvaccinated)				
One vaccination	1.75(0.23-13.4)	3.01**(1.22-7.46)	4.32***(2.3-8.11)	2.78***(2.14-3.62)
Two vacinations	2.64***(1.39-5.0)	2.14***(1.44-3.18)	2.34***(1.4-3.92)	2.23***(1.78-2.79)
≥three vaccinations	1.33(0.62-2.86)	1.12(0.81-1.56)	0.95(0.69-1.32)	0.66***(0.57-0.78)
**Prior comorbidities**				
Chronic heart diseases§	0.95(0.37-2.44)	1.31**(1.04-1.65)	0.95(0.75-1.19)	1.34***(1.21-1.49)
Chronic lung diseases§	1.06(0.60-1.85)	1.38***(1.13-1.67)	1.57***(1.26-1.95)	1.13**(1.01-1.28)
Impaired immune system§	2.63***(1.57-4.4)	1.64***(1.29-2.09)	1.59***(1.24-2.03)	1.36***(1.17-1.57)
Chronic liver disease§	1.31(0.36-4.77)	2.40***(1.63-3.52)	1.17(0.66-2.07)	1.38(0.89-2.15)

Chronic renal disease[§]	1.12(0.49-2.57)	1.23(0.92-1.65)	1.75***(1.30-2.36)	1.46***(1.25-1.69)
Diabetes[§]	1.55*(0.99-2.42)	1.33***(1.11-1.60)	1.02(0.83-1.26)	1.01(0.91-1.13)
Morbid obesity[§]	1.36(0.83-2.22)	1.15(0.90-1.47)	0.97(0.68-1.37)	0.85(0.65-1.12)
Neuromuscular diseases[§]	1.87(0.70-5.05)	1.55*(0.99-2.42)	1.42(0.85-2.37)	1.04(0.73-1.48)
Hypertension[§]	0.92(0.55-1.55)	0.92(0.77-1.09)	0.99(0.81-1.20)	0.89**(0.80-0.98)
Other[§]	1.32(0.83-2.10)	0.88(0.71-1.09)	1.02(0.80-1.29)	0.86**(0.75-0.98)
Stroke[¥]	8.3***(2.17- 31)	1.68**(1.07-2.66)	1.40(0.90-2.19)	1.50***(1.18-1.90)
Depression[¥]	1.38(0.43-4.47)	1.66*(0.92-2.99)	1.60(0.76-3.40)	1.01(0.63-1.61)
Psychiatric disease[¥]	0.83(0.28-2.44)	0.59**(0.35-1.00)	0.92(0.49-1.72)	1.36(0.93-1.99)
Cancer[¥]	2.97***(1.35-6.5)	1.48***(1.11-1.97)	1.19(0.92-1.52)	1.22***(1.08-1.39)
**ICU Admission**				
Type of ICU unit (ref. specialised ICU)				
General ICU	1.70(0.60-4.80)	1.37(0.89-2.09)	1.14(0.66-1.97)	1.41**(1.06-1.87)
Infectious ICU	1.12(0.30-4.13)	0.87(0.50-1.50)	0.67(0.34-1.30)	1.16(0.82-1.65)
Arrival route (ref. hospital ward)				
Emergency department	1.61**(1.07-2.44)	1.32***(1.11-1.58)	0.76**(0.61-0.95)	0.99(0.88-1.11)
Other unit	1.45(0.78-2.70)	1.54***(1.14-2.07)	0.73(0.47-1.14)	1.03(0.85-1.26)

Footnote: [§]from Swedish Intensive Care Register data. ¥ from National Patient Register data. VOC: Variant of concern. ICU: Intensive Care Unit. *p<0.05, **p<0.01, ***p<0.001

eTable 10. Sensitivity analysis for the association between treatment strategy, severity conditions, interventions/procedures, organ failure during ICU stay (model 2), medications administered during ICU stay (model 3) and mortality among ICU patients in Sweden in the entire cohort, *by age group*.

Variables	18-49 year	50-59 year	60-69 year	70+
	**aHR (95%CI)**	**aHR (95%CI)**	**aHR (95%CI)**	**aHR (95%CI)**
**Treatment strategy, severity conditions, interventions/procedures, organ failure (Model 2)**				
Treatment strategy (ref: without restriction)				
With restriction	22.2***(13.8-35.9)	10.7***(8.92-12.8)	8.43***(6.75-10.5)	5.14***(4.56-5.8)
Missing decision	1.32(0.60-2.91)	0.89(0.58-1.34)	1.60*(0.97-2.63)	1.21(0.93-1.58)
Severity conditions				
ARDS	0.67(0.40-1.12)	0.99(0.79-1.24)	0.81*(0.63-1.04)	0.90*(0.80-1.02)
Septic shock	1.39(0.75-2.55)	1.33**(1.03-1.71)	1.57***(1.16-2.12)	1.20**(1.00-1.44)
Intervention/procedures during ICU stay (combination) – ref. without interventions				
HFNC/CPAP	0.24*(0.05-1.06)	0.49***(0.29-0.84)	0.74(0.40-1.35)	0.90(0.71-1.13)
NIV	0.36*(0.12-1.05)	1.05(0.71-1.55)	1.20(0.72-1.98)	1.22**(1.01-1.49)
IMV	1.96**(1.07-3.56)	1.54***(1.11-2.14)	1.75**(1.12-2.72)	1.26**(1.05-1.51)
Organ failure during ICU stay				
CNS	1.69(0.76-3.74)	1.31(0.80-2.15)	1.42(0.88-2.30)	1.00(0.76-1.30)
Cardiac	2.98**(1.24-7.16)	1.03(0.69-1.52)	1.47*(0.95-2.27)	1.18(0.93-1.51)
Gastrointestinal	0.93(0.25-3.44)	1.07(0.62-1.85)	0.65(0.37-1.16)	1.31*(0.97-1.78)
Renal	1.80(0.86-3.78)	1.09(0.82-1.44)	0.71*(0.50-1.02)	1.10(0.89-1.34)
Other organ	2.09***(1.24-3.52)	1.19(0.90-1.58)	1.41**(1.02-1.96)	1.29***(1.08-1.54)
**Medication received during ICU stay (Model 3)**				

Chloroquin-phosphate	1.61(0.67-3.85)	1.55**(1.06-2.27)	1.27(0.82-1.98)	1.12(0.83-1.51)
Tocilizumab	0.80(0.36-1.79)	0.73*(0.52-1.01)	1.05(0.73-1.51)	0.79*(0.61-1.02)
Lopinavir/Ritonavir	-	-	-	16.9***(2.35-121)
Remdesivir	1.19(0.63-2.25)	0.98(0.75-1.28)	1.03(0.77-1.38)	0.97(0.82-1.14)
Baricitinib	-	1.59(0.69-3.65)	1.08(0.26-4.47)	1.25(0.71-2.18)
Steroids	0.57***(0.37-0.86)	1.03(0.87-1.23)	0.88(0.72-1.09)	0.88**(0.79-0.98)
Other drug	0.67(0.34-1.29)	0.89(0.69-1.14)	1.01(0.77-1.32)	1.03(0.89-1.20)

Footnote: VOC: Variant of concern. aHR – adjusted hazard ratio. ARDS-acute respiratory distress syndrome. HFNC-High-Flow Nasal Cannula. CPAP-Continuous Positive Airway Pressure. NIV-Non-Invasive Mechanical Ventilation. IMV-Invasive Mechanical Ventilation. CNS: Central Nervous System. *p<0.05, **p<0.01, ***p<0.001

eFigure 1. Study flowchart of study participant

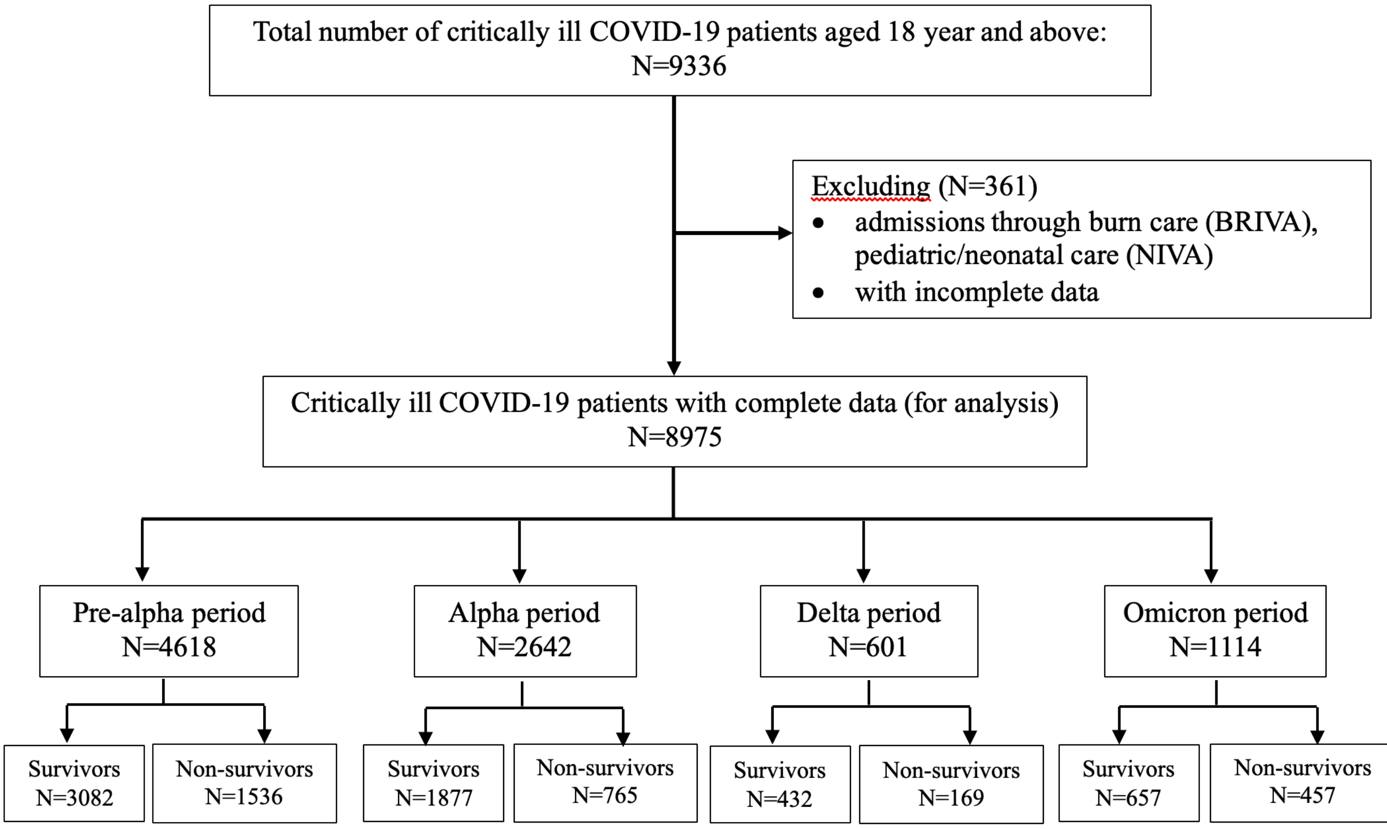

eFigure 2. Plots of Kaplan-Meier estimates of survival of COVID-19 patients admitted to ICU by variant of concern periods

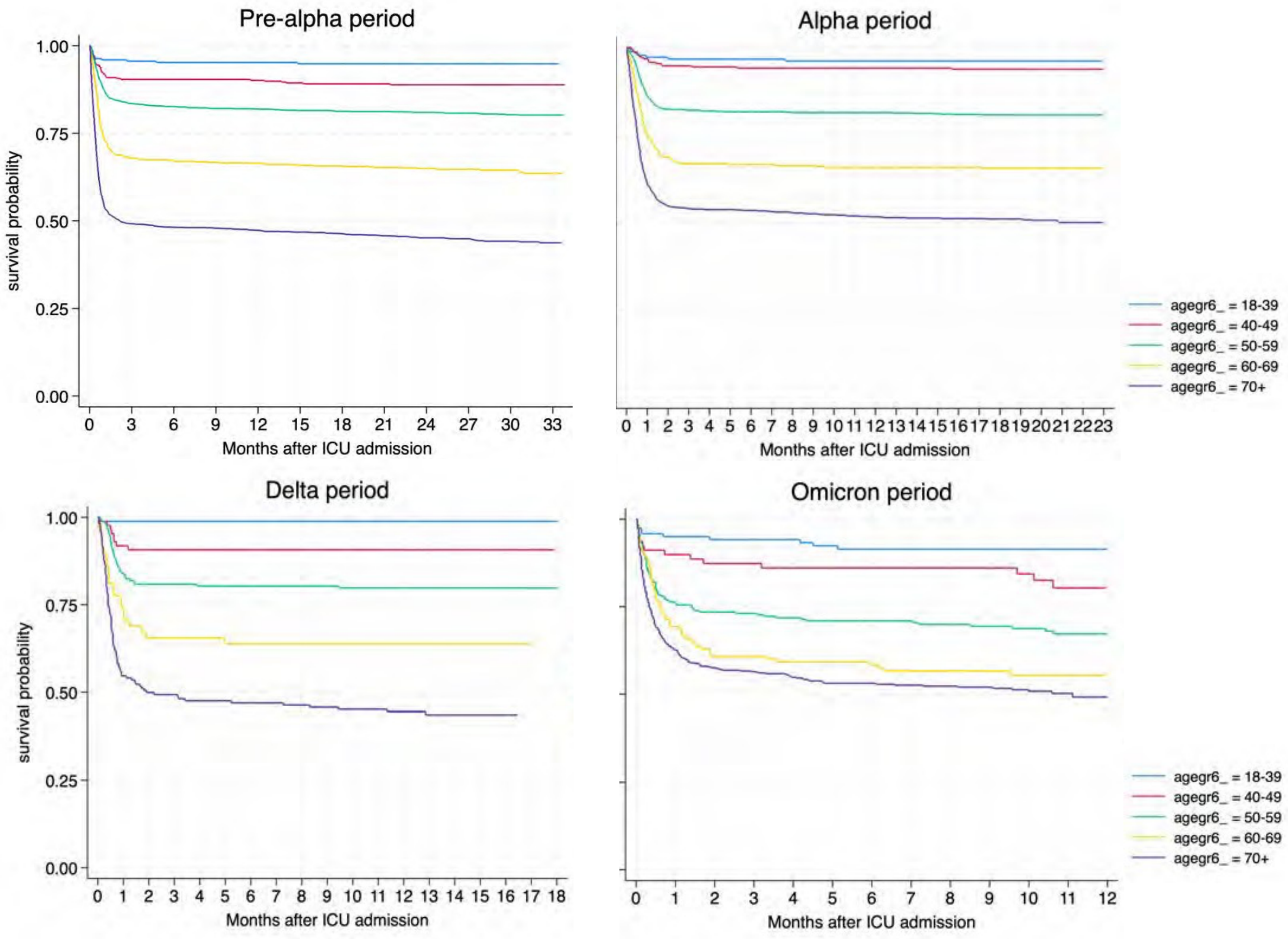

eFigure 3. COVID-19 oxygen support intervention/procedures in critical care patients across variant of concern periods.

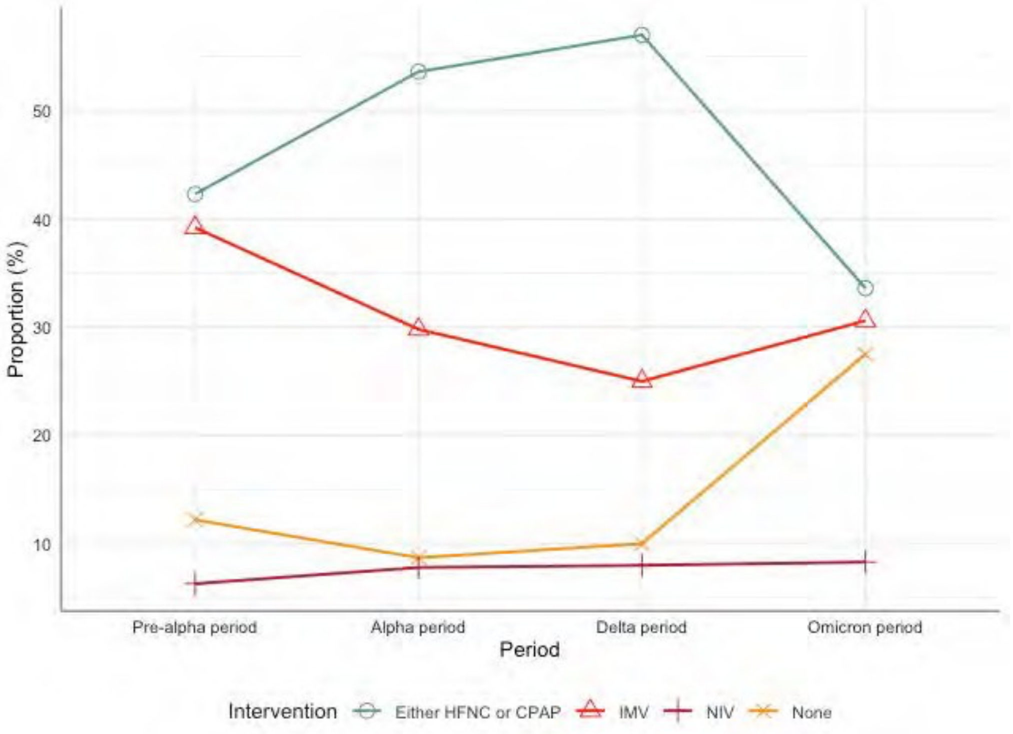

Footnote**:** IVM-Invasive Mechanical Ventilation; NIVM-Non-Invasive Mechanical Ventilation; HNFC- High-Flow Nasal Cannula; CPAP-Continuous Positive Airway Pressure

eFigure 4. COVID-19-specific medication received during ICU stay in critical care patients across variant of concern periods.

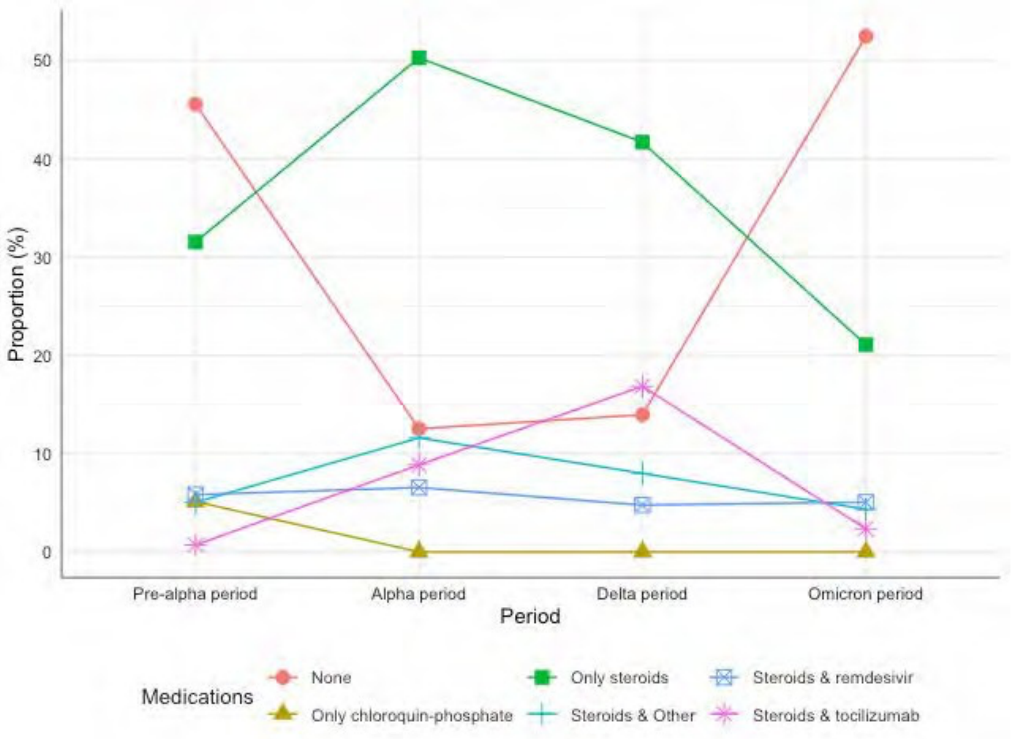

Supplement: Supplementary file 1 [file ccm-52-1194-s001.pdf]
